# Porphyrin NanoMetal-Organic Frameworks as Cancer Theranostic Agents

**DOI:** 10.3390/molecules27103111

**Published:** 2022-05-12

**Authors:** Flávio Figueira, João P. C. Tomé, Filipe A. Almeida Paz

**Affiliations:** 1 Department of Chemistry, CICECO—Aveiro Institute of Materials, University of Aveiro, 3810-193 Aveiro, Portugal; filipe.paz@ua.pt; 2 Centro de Química Estrutural, Institute of Molecular Sciences, Departamento de Engenharia Química, Instituto Superior Técnico, Universidade de Lisboa, Av. Rovisco Pais, n° 1, 1049-001 Lisboa, Portugal; jtome@tecnico.ulisboa.pt

**Keywords:** porphyrins, metal-organic frameworks, nanomaterials, theranostic, cancer treatment, cancer diagnostic

## Abstract

Metal-Organic Frameworks (MOFs) are hybrid multifunctional platforms that have found remarkable applications in cancer treatment and diagnostics. Independently, these materials can be employed in cancer treatment as intelligent drug carriers in chemotherapy, photothermal therapy, and photodynamic therapy; conversely, MOFs can further be used as diagnostic tools in fluorescence imaging, magnetic resonance imaging, computed tomography imaging, and photoacoustic imaging. One essential property of these materials is their great ability to fine-tune their composition toward a specific application by way of a judicious choice of the starting building materials (metal nodes and organic ligands). Moreover, many advancements were made concerning the preparation of these materials, including the ability to downsize the crystallites yielding nanoporous porphyrin MOFs (NMOFs) which are of great interest for clinical treatment and diagnostic theranostic tools. The usage of porphyrins as ligands allows a high degree of multifunctionality. Historically these molecules are well known for their reactive oxygen species formation and strong fluorescence characteristics, and both have proved helpful in cancer treatment and diagnostic tools. The anticipation that porphyrins in MOFs could prompt the resulting materials to multifunctional theranostic platforms is a reality nowadays with a series of remarkable and ground-breaking reports available in the literature. This is particularly remarkable in the last five years, when the scientific community witnessed rapid development in porphyrin MOFs theranostic agents through the development of imaging technologies and treatment strategies for cancer. This manuscript reviews the most relevant recent results and achievements in this particular area of interest in MOF chemistry and application.

## 1. Introduction

The development of materials with controlled structures and properties has created a plethora of prospects for disease diagnostics and therapy, particularly in oncology [1,2,3]. In recent years, much has been explored and discussed concerning the importance of the size and morphology of designed materials being highly associated with their biological performance, such as cellular uptake, cytotoxicity, biodistribution and blood circulation [4,5]. This is particularly relevant for those materials developed in the nanoscale dimension, whose unique physicochemical properties boost their biomedical applications [6,7,8,9]. Nanomaterials are fabricated into different shapes and morphologies, such as spherical nanoparticles, nanorods, nanosheets, nanodots, nanotubes, nanowires and nanocages [10,11,12,13,14,15]. This immense variety increases the biological applicability of nanomaterials where pore size and shape can embody compounds with unique abilities to treat and monitor the therapeutic effect in real-time [16,17,18].

To this end, porous nanostructures such as Metal-Organic Frameworks (MOFs), because of their porosity and high surface areas, are highly regarded for biological applications [19,20]. Recent research on MOFs, focused on the ability to control the crystallite size down to the nanometre range (NMOFs), has been opening the door to many unique properties that their micrometre counterparts do not have [21,22,23,24,25,26,27]. For instance, NMOFs are promising platforms for molecular imaging or drug delivery because of their porosity, tunable design, low toxicity, loading capacity, and intrinsic biodegradability. In addition, the inherent loading capacity endows these materials with the capability of loading and releasing different cargos, especially therapeutic agents [28,29]. These characteristics combined with the nanometer size grant enhanced cell permeation, fast cargo adsorption and access to active sites.

Besides the size and morphology of NMOFs, the judicious choice of their starting materials (metal nodes and organic ligands) further endows a high degree of multifunctionality. Linker modification (type, length, functionality, and charge) can consistently fine-tune their applicability in biomedicine by changing the crystallite morphology of NMOFs, composition and multiple physicochemical properties [30]. To this end, porphyrins are a unique class of compounds because they have a long withstanding proven application in theranostics and can be incorporated in NMOFs with relative simplicity [31,32,33,34].

Porphyrins and their various analogues (chlorins and benzoporphyrins) have distinct photophysical properties and broad absorption profiles, which span from the ultraviolet region to the near-infrared (NIR) one [35,36,37]. The most significant hallmark of these quintessential pigments imparts on reactive oxygen species (ROS) generation upon light irradiation, prompting them for therapeutic applications involving benign elements such as light and oxygen [38,39,40,41]. Porphyrins’ core, however, exhibits low water solubility for biological purposes, causing self-aggregation, and affecting the cancer treatment outcome [31]. A workaround for these disadvantages lay in their core modification with “bio”-motifs and attaching, or encapsulating, them to nanocarriers, improving their selectivity, immune tolerance, and lifetime in biological media and targeted tissues [42]. To this end, porphyrin-based nanomaterials, including micelles, liposomes, polymeric nanoparticles, peptide nanoparticles, small-molecule-nanostructures, and porphyrin NMOFs have been studied in cancer imaging and therapy [43,44]. Porphyrin NMOFs retain most of the properties of individual porphyrins, such as light-mediated generation of reactive oxygen species, especially singlet oxygen, and fluorescence properties [45]. Moreover, they can be modulated with paramagnetic metal ions for the development of imaging contrast agents for MRI (Magnetic Resonance Imaging) or PTT (Photothermal therapy) therapeutic drugs [46,47].

**Figure 1:**
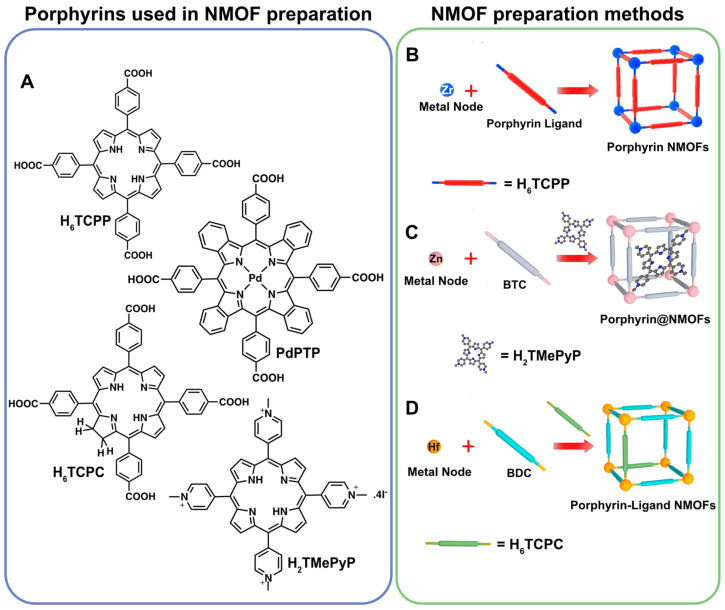
(**A**) Common porphyrin and porphyrin analogues used in the preparation of porphyrin NMOFs. (**B**) Porphyrin NMOFs preparation with a porphyrin ligand and a metal source. (**C**) Porphyrin inclusion in porous NMOFs. (**D**) Mixed ligand NMOF preparation in which porphyrins are a part of the synthesis. Reprinted/adapted with permission from Ref. [53].

The most prolific method to obtain porphyrin MOFs is to use the tetrakis(4-carboxyphenyl)porphyrin (H_6_TCPP, Figure 1A) and combine it with a wide variety of metals to achieve a set of different structures (Figure 1B–D). The most studied porphyrin MOFs are the porous coordination networks family (PCN), of which PCN-222 ([Zr_6_(µ_3_-O)_8_(TCPP)_2_] and PCN-224 ([Zr_15_(TCPP)_3_(µ_3_-O)_16_(OH)_20_(H_2_O)_4_]) are remarkable examples (Figure 1B) [48,49,50,51,52]. These NMOFs are prepared with H_6_TCPP and zirconium, while the remaining porphyrin compounds and analogues are enclosed on other common networks such as HKUST-1 and UiO-66 (Figure 1C,D). The enclosing procedures can either be achieved by supramolecular inclusion, where the porphyrin is held inside the MOF pores during its preparation (Figure 1C), or the porphyrins are used as co-ligands in the MOF preparation achieving a mixed ligand material (Figure 1D). The last method requires the porphyrin to be incorporated within the structure using the right amount so the structure and crystallinity of the NMOF are not disrupted (Figure 1C).

This review highlights the most recent advances (particularly in the last five years or so) on the multifunctionality of porphyrin NMOFs as theranostic agents. The analysis intends to show that these materials are excellent candidates for simultaneous imaging/therapy applications and that many more advancements are on the horizon in this area of research. The review is further embodied with a pedagogical nature trying to introduce to the reader many concepts and known ideas already present in the various fields that come together under the umbrella of the general subject of this manuscript.

## 2. Nano-sized Porphyrin MOFs Cancer Theranostic Modes

### 2.1. Cancer Therapy Modes

**Figure 2:**
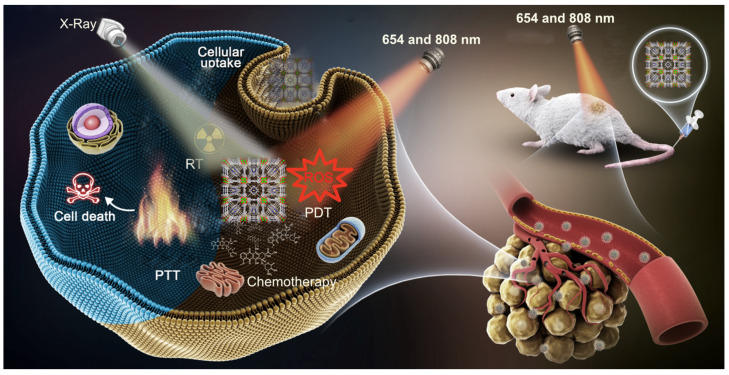
Schematic illustration of cancer therapy techniques used with porphyrin NMOFs. Reprinted/adapted with permission from Ref. [54].

Porphyrin NMOFs are tailorable theranostic platforms for cancer diagnosis and cancer treatment, including monomodal therapeutics such as photodynamic therapy (PDT), photothermal therapy (PTT), chemotherapy and radiotherapy (RT) (Figure 2).

PDT is based on the action of a photosensitiser administrated intravenously (or topically) to patients, followed by local illumination to produce ROS [55,56]. This technology exploits optical fibres or LEDs with a wavelength close to the NIR (generally in the 630–660 nm wavelength) region for deeper tissue penetration. The treatment outcome depends on the nature of the cancer cells, properties, and localisation of the tumour [57]. We note that the highly localised nature of PDT has obvious advantages because it typically causes negligible damage to the surrounding normal tissues and has minor systemic effects [58]. There is no known and obvious mechanism for acquiring resistance to PDT, which makes it a promising modality for treatment where porphyrins alone are extensively used to treat skin cancers and other non-neoplastic diseases such as macular degeneration [59,60].

PTT utilises light (generally in the 808 nm wavelength) to induce hyperthermia/thermal ablation, and porphyrin PTT agents with low fluorescence quantum yield and singlet oxygen production efficiency can achieve efficient thermal events upon NIR irradiation [61,62]. Improving the intrinsic physical properties of porphyrins for an efficient PTT requires either a shorter excited state lifetime through self-assembly of porphyrin molecules into nanoformulations, or the inclusion of certain metal cations into the porphyrin core [63]. We emphasise that it is known that porphyrin materials can be joined with gold and silver nanoparticles with localised surface plasmon resonance wavelengths to improve light absorption, ultimately enhancing the photothermal effect under light irradiation [64].

MOFs have been explored in cancer treatment as drug carriers because of their high loading capacity and excellent biocompatibility [65]. Noteworthy, since drugs commonly used in cancer treatment often cause side effects, MOF modulation can provide a clever drug delivery and release systems for more effective chemotherapy [66].

The ability of porphyrin-based NMOFs to generate toxic ROS in tumour tissues, allied to drug delivery properties, permits a controlled release of chemotherapy drugs during PDT treatment. This combined treatment enhances the therapeutic efficacy, in which lower drug doses are used while obtaining the desired antitumour effect with the additional advantage of reduced toxicity for normal cells/tissues [67].

RT is a local treatment that inflicts ionisation damage to tumour tissues in an X-ray dose-dependent method. RT efficacy is usually limited by the maximum radiation dose administered to the tumour tissues without incurring substantial injuries to the neighbouring tissues or organs [68,69]. One method to reduce X-ray doses while maintaining sufficient ionisation damage to tumours is to use heavy metals with high X-ray absorption coefficients to significantly increase the radiosensitivity difference between healthy cells and tumours [68]. This can be achieved by combining tumour-targeted porphyrin NMOFs with heavy metals such as Au and Hf that work as radio enhancers, minimising side effects to the surrounding tissues while making RT a compatible and effective treatment.

### 2.2. Cancer Imaging Techniques

**Figure 3:**
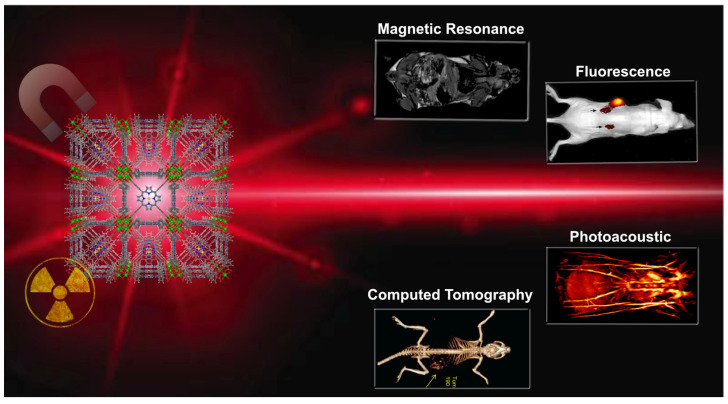
Schematic illustration of cancer imaging techniques used with porphyrin NMOFs.

Most diagnostic systems based on a single mode are limited. One of the most exciting advantages of using porphyrins NMOFs imaging agents is the possibility of using different imaging approaches in one platform, thereby significantly improving diagnostic accuracy. The most explored imaging modes of NMOF materials are fluorescence (FI), magnetic resonance, computed tomography (CT), and Photoacoustic (PAI) imaging (Figure 3).

Combining the porphyrin fluorescence and metals can lead to the generation or enhancement of contrast signals in targeted tissues. The intrinsic characteristics of porphyrin NMOFs can achieve multimode imaging-guided therapy, which can avoid the inherent limitations of single-mode imaging. This multi-mode imaging capability delivers more accurate localisation of lesions/particles and treatment guidelines. This section will present several examples of imaging methods used by porphyrin NMOFs theranostic agents.

FI is a fast, non-invasive screening method that offers high signal sensitivity based on fluorophore photon absorption and emission capabilities [70]. In vivo fluorescence imaging employs a sensitive camera to detect the fluorescence emission from fluorophores in whole-body small living animals. Fluorophores with extended emission at the NIR are generally used to avoid photon attenuation in tissues [70,71]. Porphyrins and analogues (chlorins and benzoporphyrins) are popular fluorophores and have long been incorporated into NMOFs for both in vitro and in vivo fluorescence imaging [72]. With the combination of fluorescence and photosensitivity from porphyrins, imaging-guided therapy nanosystems can be constructed based on porphyrin NMOFs to more efficiently visualise tumours [73].

MRI is a non-invasive imaging technique that rapidly differentiates tumours from other normal tissues [74]. This medical imaging technique uses a magnetic field and computer-generated radio waves to create detailed images of the organs and tissues. Contrasting with X-rays and CT, MRI does not involve hazardous ionising radiation and provides high spatial resolution and unlimited penetration depth [75]. The low MRI sensitivity can be improved by contrast agents that usually are divided into positive contrast agents (T_1_), which can reduce the longitudinal relaxation time and negative contrast agents (T_2_) that can shorten the transverse relaxation time [76]. Gadolinium and manganese-based porphyrins can be employed as T_1_ contrast agents and incorporated into porphyrin NMOFs ultimately reducing the known toxicity of some contrast agents used in clinical diagnosis and research [77,78].

CT is a computerized imaging procedure in which X-rays are focused on a patient and quickly rotated around the body, producing signals processed by the machine’s computer to generate cross-sectional images [79]. Contrast agents with high electron density are vital for CT, with gold, iodine, bismuth or gadolinium providing different densities between organs and adjunct tissues [80]. Nonetheless, the nonspecific distribution and high doses of contrast agents used to obtain decent images may induce side effects and toxicity to normal tissues [81]. A workaround for this toxicity is metal coordination into a porphyrin core, or by combining MOFs with gold nanoparticles in a composite. These approaches may diminish the overall toxicity of the contrast agent, either by lowering the administered doses or by increasing their stability and specificity.

PAI is a technology that combines optical and ultrasound imaging for the detection of endogenous or exogenous chromophores that are excited through an illumination carried out with pulsed laser light, typically in the NIR range [82]. The nonradiative release of the absorbed energy produces a micro heating and a thermoelastic expansion in the chromophore surrounding, thus generating ultrasounds [83]. The acoustic waves are detected by a transducer and transformed into a 3D-high resolution image of soft tissues. Because of the shallow penetration depth, PAI is limited to superficial organ analysis.

## 3. Porphyrin MOFs as Theranostic Agents

### 3.1. Porphyrin Inclusion into NMOFs

The first methodologies to prepare porphyrin MOF theranostic agents were indirect methods, with the porphyrin macrocycle included in the MOF network either by supramolecular entrapment or covalent attachment within the network with a tetrapodal porphyrin ligand. Both methods are accessible and straightforward ways to combine the advantages of porphyrins with NMOFs without the need to prepare pure porphyrin NMOFs.

The first approach was accomplished in 2015 with the encapsulation of tetrakis(1-methylpyridinium-4-yl)porphyrin (H_2_TMPyP) on a zinc MOF (ZnMOF) with the formula ([Zn_18_(OH)_4_(BTC)_12_(DMF)_15_]·xZnTMPyP·H_3_O_(4-4x)_]) to form the ZnTMPyP@ZnMOF [84]. ZnTMPyP@ZnMOF was later functionalized with 3-(glycidyloxypropyl)trimethoxysilane to attach Cy3-labelled (Cyanine 3) caspase-3 substrate peptide and H_2_N–PEG–folate (FA) [85]. After modification, the negative surface potential of the peptide ZnTMPyP@ZnMOF nanoprobe (amount of the peptide on material: 37.1 mg g^−1^) showed a high dispersibility and stability in water and PBS suspensions (around 30 h). A better internalization of the nanoprobe by cancer cells (HeLa cells) via folate receptor-mediated endocytosis was achieved, triggering photosensitive cell apoptosis 6.2 times higher than the porphyrin alone. The high phototoxicity results from the light induced ROS formation from ZnTMPyP in mitochondria inducing cell apoptosis with caspase-3 activation, which cleaves the Cy3-peptide graphed onto the composite surface enhancing the Cy3 fluorescence for imaging.

**Figure 4:**
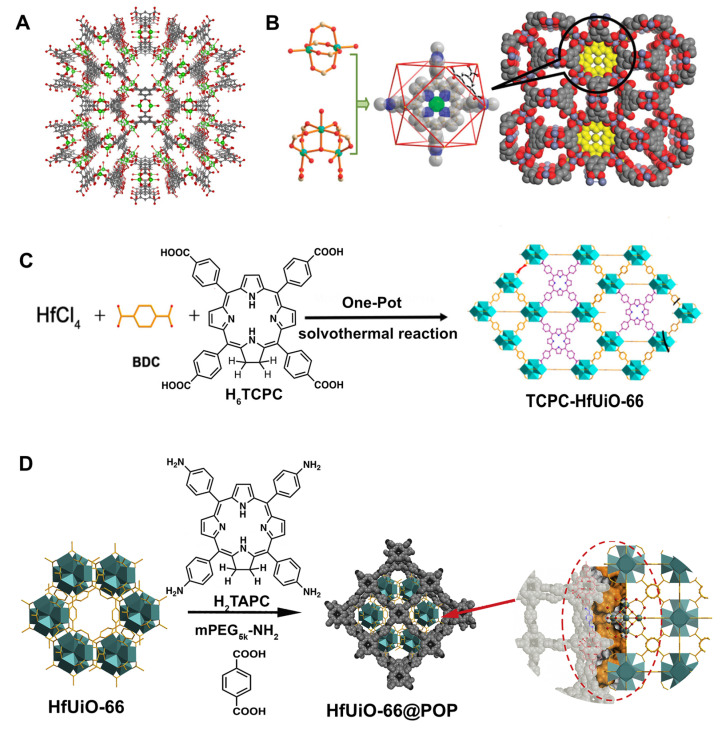
(**A**) Crystal structure of ZnMOF projected along the c axis (green: Zn, Grey: Carbon, Red: Oxygen); (**B**) The two molecular secondary building blocks in ZnTMPyP^+^@ZnMOF, Porphyrin cation located in an octahemioctahedral cage and space-filling model of ZnTMPyP^+^@ZnMOF projected along the c axis; (**C**) Synthesis of H_2_TCPC-HfUiO-66; (**D**) Synthesis of HfUiO-66@POP nanocomposite. Reprinted/adapted with permission from Refs. [84,86,87].

The second approach focused on a photoactive chlorin (H_6_TCPC) that was incorporated into the HfUiO-66 framework by a facile mixed-component one-pot strategy (Figure 4C) [86]. The rationale behind the structure choice lies in the inherent chemical stability of HfUiO-66, which displays high tolerability to accommodate geometrically different ligands in a single structure. Hafnium enhances the intersystem crossing of singlet-to-triplet by weakening spin prohibition because of the relatively sizeable spin-orbit coupling constant, which is beneficial for PTT and X-ray attenuating ability for CT imaging. The chlorin spatial arrangements and photochemical properties within H_2_TCPC-HfUiO-66 afforded the best of both worlds with enhanced multimodal-imaging-guided MRI and PTT antitumour therapy. H_2_TCPC-HfUiO-66 has an impressive anticancer activity against H22 tumour-bearing mice in vivo, with a tumour inhibition growth rate above 90%. In addition, this nanomaterial showed a high photothermal conversion efficiency, favourable photostability, and biocompatibility, being used in trimodal CT/TI/PAI as contrast agents. The success of the implementation of this material as a theranostic agent, with stronger fluorescent MOFs and higher quantum yield when compared with H_6_TCPC, was ascribed to the periodicity and regularity of chromophore distribution within the MOF framework, which is beneficial due to the increased intramolecular distances between chromophores avoiding the self-quenching effects.

Zheng and collaborators used a similar rationale to design a multifunctional platform with comparable therapy and imaging capabilities to the previous work [87]. While similar structural resemblances can be found in the starting materials (HfUiO-66 and the tetraaminophenyl chlorin, H_2_TAPC), the group employed a different approach, including HfUiO-66 within a chlorin porous organic polymer (POP) (Figure 4D). The strong extinction coefficient of chlorin compared to porphyrins at the lowest-energy Q band promoted an efficient light-harvesting ability. Consequently, both ROS and heat generation achieved a much higher antitumoural activity (tumour inhibition rate up to 88.4%) evaluated in U14 tumour-bearing Kunming strain mouse. The HfUiO-66@POP system displays good stability, biocompatibility, and high photothermal conversion efficiency.

These systems showed that porphyrins could be included in MOFs using innovative and straightforward methods for the preparation of theranostic agents with outstanding performances.

### 3.2. Porphyrin NMOFs

**Figure 5:**
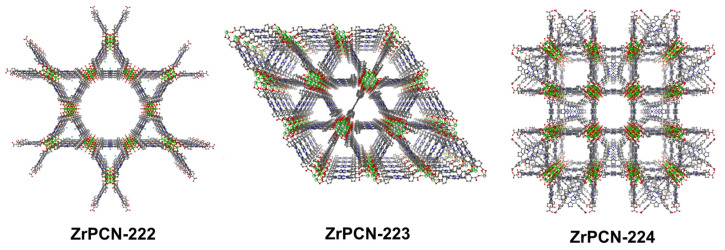
Porous coordination polymers (PCN) used as cancer theranostic agents.

The most iconic and commonly used porphyrin porous coordination networks (PCNs) are PCN-222, PCN-223 and PCN-224 (Figure 5).

**Figure 6:**
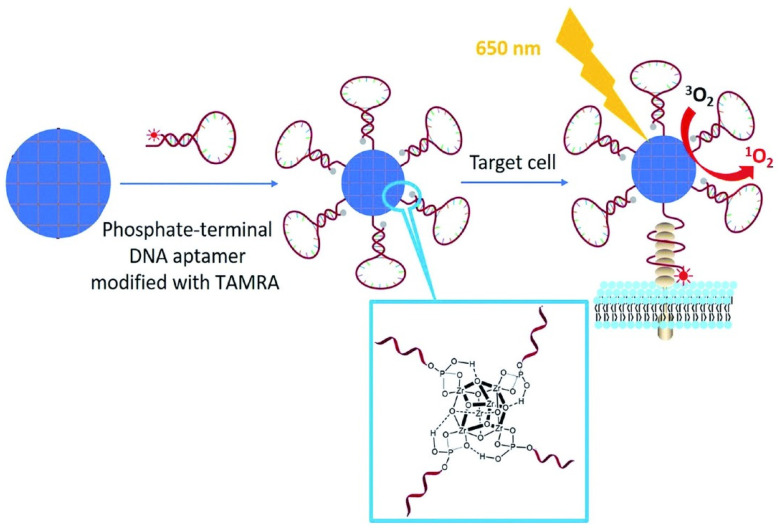
Illustration of phosphate-terminal DNA aptamer conjugation to a ZrPCN-222-P-DNA for target-induced imaging and photodynamic therapy. Reprinted/adapted with permission from Refence [89].

These are the most prolific theranostic NMOF platforms prepared to date: these materials can be integrated into image-guided therapy systems with tunable compositions for bioimaging, suitable sizes and shapes for bio transfer, proper channels and pores for drug loading, and are intrinsically biocompatible. For instance, a biocompatible nanoscale version of ZrPCN-222 was prepared from a microemulsion [88]. The high porphyrin content (59.8%) allows the use of concurrent efficient fluorescent imaging and PDT. The one-dimensional channel of the NMOF provides a high doxorubicin loading and pH-response smart release for chemotherapy. The fluorescence guiding of the chemotherapy-and-PDT dual system is confirmed by the concentration of NMOF at cancer sites after laser irradiation and doxorubicin release, while low toxicity is observed in normal tissues. Using the same MOF Liu and collaborators employed a phosphate-terminal aptamer modified with 5-carboxytetramethylrhodamine (TAMRA) and anchored to the ZrPCN-222 particles through strong coordination between the phosphate and zirconium (Figure 6) [89]. These materials ZrPCN-222-P-DNA were tested for target-induced bioimaging and photodynamic therapy.

The target-induced imaging was achieved because of the structural change of the aptamer upon binding with the complementary DNA (c-DNA) and hybridisation to form a double-stranded DNA target that enhances TAMRA fluorescence (Figure 6). This system exhibited an excellent target-induced imaging ability when incubated with HeLa cells. A similar system built with a random DNA sequence showed no target ability towards HeLa cells. On the other hand, the ZrPCN-222-P-DNA specificity towards HeLa cells increased the PDT efficiency significantly reducing cell viability to 48% and 17% when treated at concentrations of 100 μg mL^−1^ and 200 μg mL^−1^.

In 2018, gadolinium-porphyrin spherical MOF nanoparticles were conjugated with folic acid (FA), and their theranostic capabilities were tested against HepG2 cells and embryonic and larval zebrafish [90]. The photodynamic therapeutic effects of FA-MOF nanoparticles were explored in transgenic zebrafish with doxycycline-induced hepatocellular carcinoma. As described before the FA-derived MOF nanoparticles target specific cancer cells that over-express folate receptors (HeLa cells) enhancing their internalisation via folate receptor-mediated endocytosis. These nanomaterials showed low biotoxicity, emitted bright red fluorescence, and exhibited an excellent MRI contrast and PDT treatment capabilities both in vitro and in vivo.

**Figure 7:**
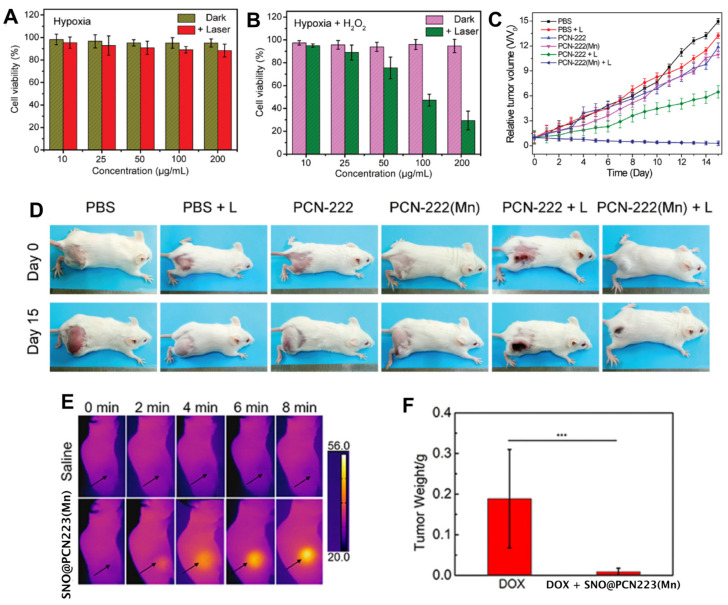
MTT assay of the 4T1 cell viability after incubation with different concentrations of ZrPCN-222(Mn) and irradiation by a 660 nm laser for 8 min under (**A**) hypoxic conditions without H_2_O_2_ and (**B**) under hypoxia in the presence of H_2_O_2_ (100 μM). (**C**) Time-dependent relative tumour volumes and mice body weights for different treatment groups. (**D**) Photographs of mice before treatment and on the 15th day after treatment. (**E**) in vitro IR thermal photos of saline and SNO@ZrPCN-223(Mn) under NIR laser irradiation. (**F**) Tumour weight after DOX chemotherapy and SNO@ZrPCN-223(Mn) combined with DOX chemotherapy (*** *p* < 0.001). Reprinted/adapted with permission from Refs. [91,92].

Meie and collaborators prepared ZrPCN-222 with MnTCPP, ZrPCN-222(Mn), having an excellent dispersibility of manganese in the open framework while the high water affinity of the channel embodied the material with a high longitudinal relaxivity [91]. This feature allied to the excellent catalytic conversion of endogenous hydrogen peroxide into oxygen improved tumour hypoxia PDT and MRI guidance (Figure 7A,B). The intravenous injection of ZrPCN-222(Mn) into tumour-bearing mice provided good tumour contrast and growth inhibition upon single-laser irradiation (660 nm laser) (Figure 7C,D). These findings demonstrate that the simple addition of Mn to the porphyrin core in ZrPCN-222 can be a practical approach to construct an O_2_ self-supplementing PDT nanoplatform with excellent T_1_-weighted MRI effective diagnosis and treatment of hypoxic tumours.

Zhang and collaborators prepared a manganese ZrPCN-223 and loaded *S*-nitrosothiol (SNO) into ZrPCN-223(Mn) nanoparticles to generate nitric oxide by heating [92]. The insertion of Mn ions into the porphyrin core rendered the NMOFs with strong T_1_-weighted MR contrast capacity and high photothermal conversion (Figure 7E). SNO is a type of heat-unstable nitric oxide donor, that upon PPT initiation and temperature increase decomposes to release NO. Tumour growth in mice injected with the SNO@ZrPCN-223(Mn) was inhibited upon NIR irradiation, validating the efficiency of in vivo nitric oxide release and photothermal therapy. Moreover, SNO@ZrPCN-223(Mn) PTT treatment followed by DOX chemotherapy exhibited higher tumour inhibition efficiency than DOX chemotherapy without the PTT treatment (Figure 7F). The role of NO in tumour therapy depends on the concentration and duration of its action within the tumour cells: in the right concentrations, it affects cancer initiation and enhances cell progression, tumour blood flow, angiogenesis, metastasis, cell death, and tumour suppression [93]. Zhou and co-workers developed a porphyrin palladium NMOF (PdNMOF) with a high dispersion of the metals and with enhanced hydrogen loading for tumour-targeted (PAI)-guided hydrogenothermal therapy [94]. The porphyrin used in this system was tetrakis(4-pyridyl)-porphyrin (H_2_TPyP), and structural characterisation of the NMOF was scarce and elusive, essentially because of the nanometer profile that does not allow single-crystal characterisation. The hydrogenated PdMOF (PdHMOF) showed, however, a sustained hydrogen release profile over five days and a high PTT effect combined with PAI performance.

Manganese porphyrins versatility promoted the development of high-performance theranostic platforms that find application in three main imaging modes, MRI/CT/PAI, and two treatment ones, PTT and RT. This was achieved with the hafnium version of HfPCN-222 coated with FA to enhance the cancer-targeting efficacy [95]. Significant tumour growth inhibition was achieved in mouse cancer models without damage to other organs.

**Figure 8:**
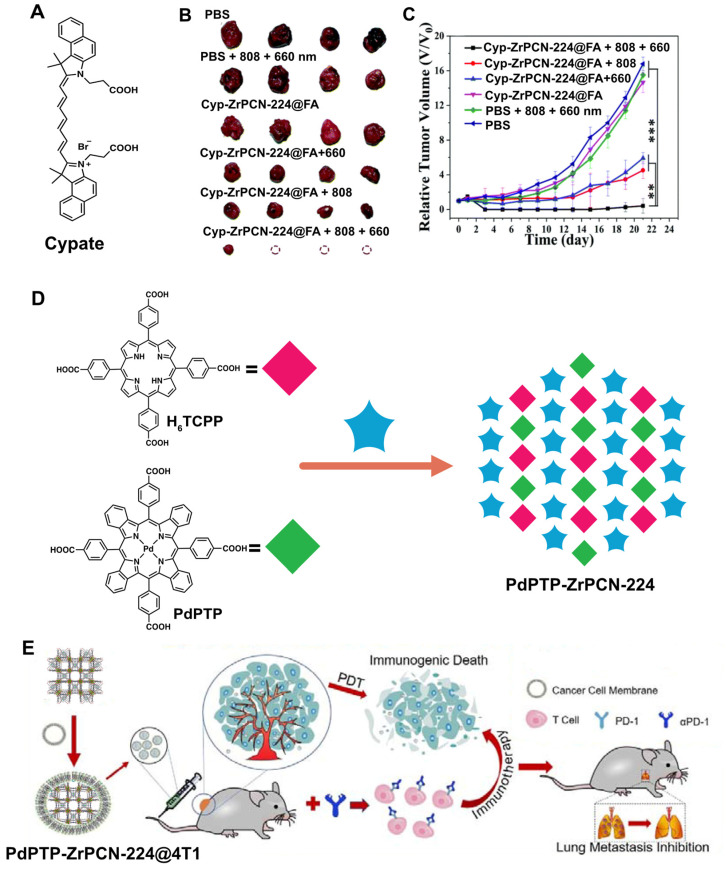
(**A**) Organic compound used to prepare Cyp-ZrPCN-224. (**B**) photos of ex vivo tumours with different treatments with Cyp-ZrPCN-224 after 21 days. (**C**) Relative tumour volumes of six groups of mice that received different Cyp-ZrPCN-224 (15 mg kg^−1^) treatments. (**D**) Schematic illustration of the synthesis and application of PdPTP-ZrPCN-224. (**E**) The PDT and αPD-1 checkpoint blockade combined therapeutic effects of PdPTP-ZrPCN-224@4T1. Reprinted/adapted with permission from Refs. [96,97]. (Data are presented as the mean ± SD (*n* = 4). **, *p* < 0.01; ***, *p* < 0.001).

Over time, these systems progressed in applicability and complexity with engineered structures with defects to include multiple ligands into the same network integrating the co-ligand functionality into the final porphyrin NMOF. This was performed by combining cypate (Cyp, Figure 8A) and porphyrin within the same NMOF structure to perform FI/PAI/PTI trimodal imaging-guided and PDT/PTT therapy modes in a single material [96]. The engineered Cyp-ZrPCN-224 was coated with FA to increase tumour tissue targeting and multimodal cancer phototheranostics. The PTT effect was concentration-dependent, and a lower cell survival rate was observed for the dual-laser treatment, which had an IC_50_ of 45.9 μg mL^−1^. Mice bearing 4T1 tumours were studied in terms of tumour volume after Cyp-ZrPCN-224@FA administration and combined 660 nm (0.2 W cm^−2^, 5 min.)/808 nm (1 W cm^−2^, 5 min.) laser irradiation. A total tumour suppression was observed with an inhibition rate of up to ∼97.15%, expressing the remarkable efficacy of PTT/PDT combined therapies (Figure 8B,C).

Xie and co-workers used a similar approach to include a co-ligand into the ZrPCN-224 network to boost both the PDT effect and the near-infrared (NIR) O_2_ concentration ratiometric imaging. This approach addressed a number of limitations associated with short detection wavelengths and the influence of self-concentrations. This was possible by doping ZrPCN-224 with an extended π-conjugated palladium porphyrin (Pd-tetrakis(4-methoxycarbonylphenyl)-tetrabenzoporphyrin) (PdPTP, Figure 8D) [97]. 4T1 cell membrane extraction and PdPTP-ZrPCN-224 coating yielded a cloaked nanomaterial (PdPTP-ZrPCN-224@4T1) capable of targeting in vivo solid tumours with high efficiency, and effectively suppressing tumour growth via PDT. The increased PDT effect of PdPTP mediated a strong immune activation by inducing cancer immunogenic cell death (Figure 8E).

Porphyrin NMOFs guarantee a high photosensitiser loading capacity and prevent the self-quenching effect observed for porphyrin molecules. There are still obstacles associated with the high glutathione (GSH) content in tumour tissues that depletes ^1^O_2_ and reduces the PDT efficiency. GSH is the most abundant antioxidant in human bodies, which can eliminate ROS and protect cells from acute toxicity, as well as repair damaged biomolecules [98]. The GSH concentration in cancer cells is often higher than that in normal cells making it one of the main obstacles for ROS-induced cell killing.

**Figure 9:**
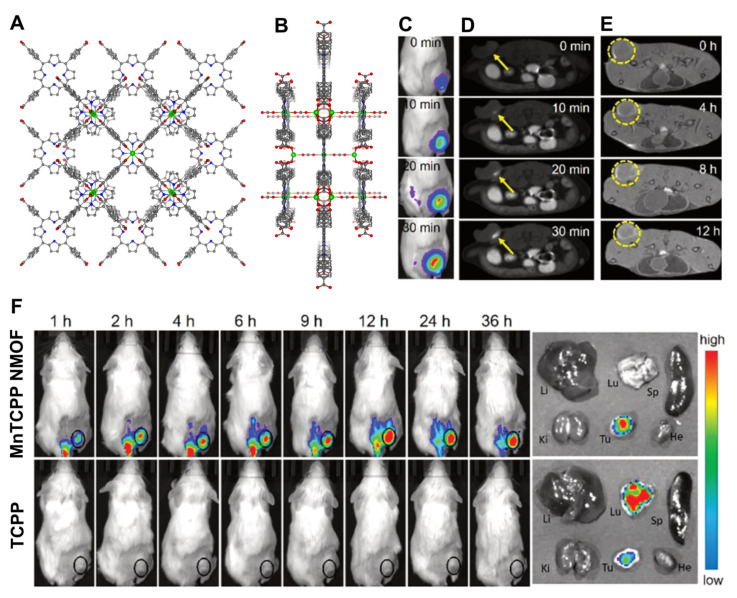
(**A**) Front and (**B**) side views of the Mn(III)TCPP NMOF. (**C**) Fluorescence and (**D**) T_1_ contrast signals in tumour sites by intra-tumoural injection of Mn(III)TCPP NMOF within 30 min. (**E**) In vivo MRI signal after intravenous injection with Mn(III)TCPP NMOF. (**F**) Fluorescence imaging of mice over time by intravenous injection of Mn(III)TCPP NMOF and tissue imaging at 36 h post-injection. Reprinted/adapted with permission from Ref. [99].

To overcome this, the excess acid and GSH in the tumour microenvironment can be surpassed with a redox reaction between Mn(III) and GSH, with the concomitant formation of glutathione disulphide and Mn(II). The consumption of GSH and in situ formation of Mn(II) avoids the antioxidant nature of GSH and the consequent ^1^O_2_ depletion. This concept was employed as two distinct approaches. One uses a custom-made Mn(III)TCPP NMOF prepared by way of a one-pot method (Figure 9A,B), being inert in ROS generation and exhibiting an absence of fluorescence [99]. However, the presence of glutathione (GSH) inside the cells promoted the destruction of the network into in Mn(II) and Mn(III)TCPP, enhancing the MRI and the fluorescent imaging capabilities (Figure 9C–F). More importantly, the GSH-regulated Mn(III)TCPP release could allow a controlled ROS generation under irradiation, which avoided side effects such as inflammation and damage to normal tissues.

The second approach combined MnO_2_ nanoparticles with PCN-222 to construct an MnO_2_-coated porphyrin MOF with GSH-activated MRI-guided enhanced photodynamic- and chemotherapies [100]. The preparation of PCN-222 (72 nm particles) combined with poly(allylamine hydrochloride) and MnO_2_ uniform layers around the NMOF allowed the development of a theranostic nanoplatform with high biocompatibility (no cytotoxicity at concentrations of 400 μg mL^−1^). As for the previous material, the PDT enhancement is attributed to the GSH elimination effect by the MnO_2_ layer, which simultaneously permitted GSH-responsive T_1_-MRI and the controlled release of DOX.

Investigation of the efficiency of this PCN-222 composite on 4T1 tumour-bearing mice showed a very high T_1_-MRI relaxation time after one hour of administration, exhibiting a fast depletion of GSH by MnO_2_. Notably, the groups treated with PDT and chemotherapy presented the most effective inhibition of tumour growth. Both systems proved to be efficient in PDT by combining controlled ROS generation and GSH depletion after precise MRI imaging. Interestingly, the first approach shows a much simpler system in terms of preparation, further achieving dual-mode imaging. On the other hand, the second system only vehiculates to a single-mode imaging system with a controlled release chemotherapy treatment component.

**Figure 10:**
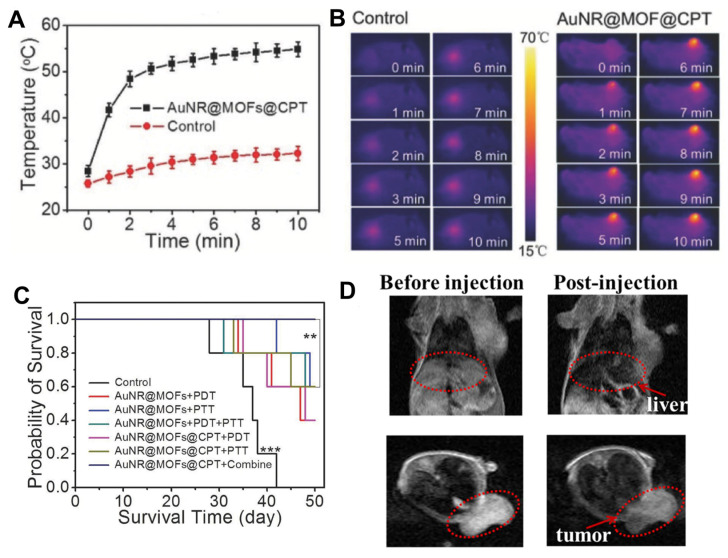
(**A**) Temperature change curve with AuNR@MOF@CPT of tumour tissues as a function of irradiation time. (**B**) in vivo thermal images of the mice after intravenous injection of PBS and AuNR@MOFs@CPT with 808 nm laser irradiation. (**C**) Survival curves of tumour-bearing mice after different treatments with AuNR@MOF@CPT (*n* = 5, ** *p* < 0.01 and *** *p* < 0.01 were calculated by a Student’s *t*-test). (**D**) Fe_3_O_4_@C@PCN-222 T_2_-weighted MRI of tumour-bearing mice in the coronal plane (upper) and the axial plane (lower). A red dot line marked the liver region and tumour region. Reprinted/adapted with permission from Refs. [101,102].

Other materials can be combined with porphyrin NMOFs to increase MRI and PTT treatments (besides just the use of manganese and hafnium). For instance, Zhang and co-workers fabricated a core-shell gold nanorod@PCN-224 nanocomposite (AuNR@MOF) in which porphyrin NMOFs were grown on the surface of gold nanorods, and a large amount of camptothecin (CPT) was loaded into the shell of AuNR@MOF [101]. When AuNR@MOF@CPT was irradiated with an 808 nm laser, the nanocomposites exhibited the PTT effect, and the temperature of the AuNR@PCN-224@CPT aqueous solution increased to 51.8 °C in around 2 min., and up to 73.4 °C in just 10 min. (Figure 10A,B). The increased temperature promoted the controlled release of CPT. When irradiated with a 660 nm laser, the therapeutic effect was mainly associated with PDT arising from the porphyrin ligands present in the MOF structure. The composite PDT effect was much stronger than that of pure porphyrin, with the results indicating that the gold nanorod core played a decisive role in the absorption of excited light and the electromagnetic field, thus enhancing the PDT effect. Combined with the chemotherapy effect of the loaded CPT, the AuNR@MOFs exhibited a synergistic antitumour effect of PDT and PTT under irradiation with lights of two different wavelengths, and the tumours of the treated mice almost disappeared within 50 days of treatment (Figure 10C).

Integrated systems with gold nanoparticles and porphyrin NMOF show tremendous promise in the theranostic field. The fabrication of low-cost materials within a single high-efficiency theranostic platform remains, however, a challenge [103]. The PDT oxygen dependency makes it less efficient under hypoxic conditions on solid tumours, and the generation of ROS has a narrow effective function range. PTT with gold, manganese and other nanomaterials such as graphene, graphene oxide and polypyrroles have shown, conversely, promising results in this field [104]. In general, incorporating MRI and PTT modes in a single material increases the cost and the overall complexity. A simple workaround replaces the gold nanorod core described above with a cluster of Fe_3_O_4_ nanoparticles encapsulated in a carbon shell (Fe_3_O_4_@C). Zhang and collaborators employed this approach using superparamagnetic iron oxide nanoparticles (SPIONs) [102]. These are efficient imaging agents because they shorten the transverse relaxation (higher T_2_-MRI efficiency, Figure 10D) and have excellent biocompatibility. The composite was prepared in situ with PCN-222 growth over Fe_3_O_4_@C. The T_2_-weighted MRI/fluorescence imaging-guided PTT and PDT dual-therapy was tested on tumour-bearing mice demonstrating a high tumour accumulation and efficient ablation with similar laser irradiation at 808 and 655 nm.

A nanoscale iron porphyrin NMOF was prepared with the same concept in mind, bearing the same T_2_-weighted MRI/fluorescence imaging-guided and PTT and PDT dual-therapy [105]. The nanoparticles were coated with BSA, using carbodiimide crosslinker chemistry methods (EDC/NHS) to achieve a higher accumulation at the tumour sites and an in vivo increasing circulation time for active hypoxic tumour targeting. In vitro, ROS detection and photothermal temperature increase revealed that these nanoplatforms could exhibit a great PDT effect in tumour cells under hypoxic conditions. Unfortunately, the structure and molecular formula of the iron porphyrin MOF remain elusive and, therefore, it is highly complex to conclude anything about the structure-affinity of these nanoparticles.

### 3.3. Porphyrin NMOF Sheets

**Figure 11:**
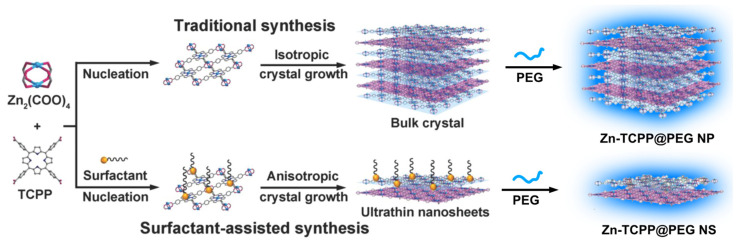
NMOF sheets general preparation method and PEG coating. Reprinted/adapted with permission from Ref. [106].

Nanoscale MOFs with porous structures and inherent biodegradability are attractive nanomedicine platforms. In addition to conventional particulate NMOFs, two-dimensional (2D) NMOFs are emerging as a unique type of material for nanomedicine applications. Their preparation was developed in 2015 with a surfactant-assisted synthesis (polyvinylpyrrolidone, PVP) with different metals (Zn, Cu, Cd, or Co and H_6_TCPP [106]. The surfactant plays a key role in the controlled growth of the MOF crystals, leading to anisotropic growth and the formation of ultrathin MOF nanosheets (Figure 11).

Several research papers reported the usage of these methods to prepare 2D porphyrin NMOF sheets for various applications [31,50,107,108,109,110]. Their use in theranostic increased exponentially. A recent example shows the comparison of a 2D NMOF composed of Zn^2+^ and H_6_TCPP to their particulate counterparts. The 2D NMOFs exhibited an increased DOX loading capacity and enhanced light-triggered singlet oxygen production [111,112]. In addition, the drug-loaded 2D NMOFs could be easily labelled with ^99m^Tc for single-photon emission computer tomography imaging (SPECT), being an indication of an efficient tumour homing of these materials upon intravenous injection. This system arises as a remarkable tool for the combined in vivo antitumour effect and real-time imaging, with the material further showing efficient biodegradation and rapid renal clearance. This research clearly shows the advantages of 2D NMOF sheets in singlet oxygen generation. Nonetheless, using these materials with different metals permits the modulation of their therapeutic effect. For instance, replacing zinc with copper in the preparation of the nanosheets improves the NIR absorption because of the *d*-*d* energy band transition of Cu^2+^ and the ultrathin characteristic translating into excellent photothermal performance and MRI applications [68]. The singlet oxygen generation inherent to H_6_TCPP is maintained, thus extending the treatment possibilities in a single nanomaterial. The 2D NMOF sheets were prepared with a mean thickness of 5.1 nm and were employed in imaging-guided phototherapy. Interestingly only a marginal inhibition of tumour growth was observed with one single treatment, PTT or PDT, in nude mice bearing Saos-2 tumours. In contrast, other mice groups treated with the combination of PTT and PDT (PTT: 808 nm laser (1.0 W cm^−2^ for 10 min) or the PDT: 660 nm laser (10 mW cm^−2^ for 30 min)) had their tumours retrogressed. This work showed how the synergy between the multitude of the porphyrins’ benefits could introduce into these materials, permitting the use of simultaneous PDT, PTT and external magnetic fields for MR imaging of cancers in vivo.

## 4. Conclusions

Much remains to be undertaken to transpose porphyrin NMOFs into clinical practice. Looking into the bigger picture, NMOF materials as theranostic agents are a subject of research in their infancy. However, the potential of theranostic nanoparticles is progressively gaining the attention of major clinical players and biomedicine in general.

Porphyrin NMOF probes have several advantages when compared to conventional approaches, where diagnostic and treatment procedures are frequently separated, and while there are many challenges to overcome the synthetic processes, material structures and corresponding functional modifications are described in detail, highlighting porphyrin NMOFs as promising theranostic tools for cancer treatment and diagnostic with good performances. In fact, NMOFs, show unique properties, such as accelerated adsorption/desorption kinetics, fast accessibility to the internal active sites and above all suitable sizes for biomedical applications where the nanometric size allows NMOF particles to enter living cells via the cells’ endocytic mechanisms or alternatively by passive penetration of the lipid bilayer.

With further understanding of the synthetic mechanism of NMOFs preparation, a few methods were established to command the size of NMOFs by carefully isolating the nucleation and growth process during the synthesis. In this regard, the microemulsion methods offer the greatest possibility of achieving success, nonetheless, most attempts to pursue one method to prepare uniform NMOFs with all kinds of structures are still elusive. While the synthetic preparation of these materials, especially with porphyrin ligands, remains a challenging aspect to overcome in years to come, some examples of NMOF theranostic agents described in the literature also bypass critical steps in thoroughly examining the relationship between their intrinsic properties and the final applications. There are still a few critical aspects that need to be fully addressed in this subject. Issues ranging from toxicity to efficacy and precision therapy to efficient biomarkers are still unclear and require focused research and attention. Furthermore, the structure affinity relating to size, shape, and structure and how these characteristics influence the therapeutic efficacy in vivo also needs attention in years to come. The wide variety of modalities that can be combined into a single material is undoubtedly a major advantage of these materials and preparation methods are becoming more efficient streamlining the obtention of new porphyrin NMOFs capable of application as theranostic agents. Despite all these challenges and considering that most of the developments around porphyrin NMOF theranostic agents have occurred in the last 5 years, we anticipate most of these limitations to be exceeded in years to come. Theranostics have unparalleled value in diagnosing and treating cancers and the constant evolution of techniques engineering, and porphyrin NMOFs can have a tremendous impact on cancer theranostic treatment and diagnosis.

## Data Availability

Not applicable.

## Abbreviations

AuNR	Gold nanorods
BTC	Benzene 1,3,5-tricarboxylate
Cy3	Cyanine 3
DOX	Doxorubicin
CPT	Camptothecin
CT	Computed Tomography
EDC	1-Ethyl-3-(3-dimethylaminopropyl)carbodiimide
FA	Folate
FI	Fluorescence imaging
GSH	Glutathione
H_2_TMPyP	tetrakis(1-methylpyridinium-4-yl)porphyrin
H_2_TPyP	tetrakis(4-pyridyl)porphyrin
H_2_TAPC	tetrakis(4-aminophenyl)chlorin
H_6_TCPC	tetrakis(4-carboxyphenyl)chlorin
H_6_TCPP	tetrakis(4-carboxyphenyl)porphyrin
HKUST	Hong Kong University of Science and Technology
LED	Light-emitting diode
MRI	Magnetic Resonance Imaging
MOF	Metal-Organic Frameworks
MTT	3-(4,5-dimethylthiazol-2-yl)-2,5-diphenyltetrazolium bromide
NIR	Near-Infrared
NMOF	nano Metal-Organic Frameworks
NHS	*N*-hydroxysuccinimide
PdPTP	Palladium-tetrakis-(4-carbonylphenyl)-tetrabenzoporphyrin
PDT	Photodynamic therapy
PTT	Photothermal therapy
PAI	Photoacoustic imaging
PCN	Porous Coordination Polymer
ROS	Reactive oxygen species
RT	Radiotherapy
SPECT	Single-photon emission computer tomography
SNO	*S*-nitrosothiol
SPION	Superparamagnetic iron oxide nanoparticles
TAMRA	5-carboxytetramethylrhodamine
UiO	Universitetet i Oslo

## References

[1] Pandey A.P., Girase N.M., Patil M.D., Patil P.O., Patil D.A., Deshmukh P.K. (2014). Nanoarchitectonics in cancer therapy and imaging diagnosis. J. Nanosci. Nanotechnol..

[2] Brigger I., Dubernet C., Couvreur P. (2012). Nanoparticles in cancer therapy and diagnosis. Adv. Drug Delivery Rev..

[3] Minelli C., Lowe S.B., Stevens M.M. (2010). Engineering nanocomposite materials for cancer therapy. Small.

[4] Senapati S., Mahanta A.K., Kumar S., Maiti P. (2018). Controlled drug delivery vehicles for cancer treatment and their performance. Signal Transduct Target Ther.

[5] Barbosa J.S., Mendes R.F., Figueira F., Gaspar V.M., Mano J.F., Braga S.S., Rocha J., Almeida Paz F.A. (2020). Bone Tissue Disorders: Healing Through Coordination Chemistry. Chem. Eur. J..

[6] Makvandi P., Wang C.y., Zare E.N., Borzacchiello A., Niu L.n., Tay F.R. (2020). Metal-based nanomaterials in biomedical applications: Antimicrobial activity and cytotoxicity aspects. Adv. Funct. Mater..

[7] Liu W.L., Zou M.Z., Qin S.Y., Cheng Y.J., Ma Y.H., Sun Y.X., Zhang X.Z. (2020). Recent advances of cell membrane-coated nanomaterials for biomedical applications. Adv. Funct. Mater..

[8] Abd Elkodous M., El-Sayyad G.S., Abdelrahman I.Y., El-Bastawisy H.S., Mohamed A.E., Mosallam F.M., Nasser H.A., Gobara M., Baraka A., Elsayed M.A. (2019). Therapeutic and diagnostic potential of nanomaterials for enhanced biomedical applications. Colloids Surf. B.

[9] Figueira F., Barbosa J.S., Mendes R.F., Braga S.S., Almeida Paz F.A. (2021). Virus meet metal-organic frameworks: A nanoporous solution to a world-sized problem?. Mater. Today.

[10] Elahi N., Kamali M., Baghersad M.H. (2018). Recent biomedical applications of gold nanoparticles: A review. Talanta.

[11] Soleymaniha M., Shahbazi M.A., Rafieerad A.R., Maleki A., Amiri A. (2019). Promoting role of MXene nanosheets in biomedical sciences: Therapeutic and biosensing innovations. Adv. Funct. Mater..

[12] Chen J., Meng H., Tian Y., Yang R., Du D., Li Z., Qu L., Lin Y. (2019). Recent advances in functionalized MnO 2 nanosheets for biosensing and biomedicine applications. Nanoscale Horiz..

[13] Simon J., Flahaut E., Golzio M. (2019). Overview of Carbon Nanotubes for Biomedical Applications. Materials.

[14] Saliev T. (2019). The Advances in Biomedical Applications of Carbon Nanotubes. C.

[15] Liu Z., Li S., Xia X., Zhu Z., Chen L., Chen Z. (2020). Recent advances in multifunctional graphitic nanocapsules for Raman detection, imaging, and therapy. Small Methods.

[16] Bhunia S., Deo K.A., Gaharwar A.K. (2020). 2D covalent organic frameworks for biomedical applications. Adv. Funct. Mater..

[17] Jung Y., Huh Y., Kim D. (2021). Recent advances in surface engineering of porous silicon nanomaterials for biomedical applications. Microporous Mesoporous Mater..

[18] Aflori M. (2021). Smart Nanomaterials for Biomedical Applications—A Review. Nanomaterials.

[19] Zhang S., Pei X., Gao H., Chen S., Wang J. (2020). Metal-organic framework-based nanomaterials for biomedical applications. Chin. Chem. Lett..

[20] Leite J.P., Rodrigues D., Ferreira S., Figueira F., Almeida Paz F.A., Gales L. (2019). Mesoporous Metal–Organic Frameworks as Effective Nucleating Agents in Protein Crystallography. Cryst. Growth Des..

[21] Mallakpour S., Nikkhoo E., Hussain C.M. (2022). Application of MOF materials as drug delivery systems for cancer therapy and dermal treatment. Coord. Chem. Rev..

[22] Lawson H.D., Walton S.P., Chan C. (2021). Metal–Organic Frameworks for Drug Delivery: A Design Perspective. ACS Appl. Mater. Interfaces.

[23] Khan N.A., Hasan Z., Jhung S.H. (2018). Beyond pristine metal-organic frameworks: Preparation and application of nanostructured, nanosized, and analogous MOFs. Coord. Chem. Rev..

[24] Li Y., Fu Z., Xu G. (2019). Metal-organic framework nanosheets: Preparation and applications. Coord. Chem. Rev..

[25] Bieniek A., Terzyk A.P., Wiśniewski M., Roszek K., Kowalczyk P., Sarkisov L., Keskin S., Kaneko K. (2021). MOF materials as therapeutic agents, drug carriers, imaging agents and biosensors in cancer biomedicine: Recent advances and perspectives. Prog. Mater Sci..

[26] Mendes R.F., Figueira F., Leite J.P., Gales L., Paz F.A.A. (2020). Metal–organic frameworks: A future toolbox for biomedicine?. Chem. Soc. Rev..

[27] Simon-Yarza T., Mielcarek A., Couvreur P., Serre C. (2018). Nanoparticles of metal-organic frameworks: On the road to in vivo efficacy in biomedicine. Adv. Mater..

[28] Zhong X.-f., Sun X. (2020). Nanomedicines based on nanoscale metal-organic frameworks for cancer immunotherapy. Acta Pharmacologica Sinica.

[29] Cai M., Chen G., Qin L., Qu C., Dong X., Ni J., Yin X. (2020). Metal Organic Frameworks as Drug Targeting Delivery Vehicles in the Treatment of Cancer. Pharmaceutics.

[30] Saeb M.R., Rabiee N., Mozafari M., Verpoort F., Voskressensky L.G., Luque R. (2021). Metal-Organic Frameworks (MOFs) for Cancer Therapy. Materials.

[31] Rabiee N., Yaraki M.T., Garakani S.M., Garakani S.M., Ahmadi S., Lajevardi A., Bagherzadeh M., Rabiee M., Tayebi L., Tahriri M. (2020). Recent advances in porphyrin-based nanocomposites for effective targeted imaging and therapy. Biomaterials.

[32] Rajasree S.S., Li X., Deria P. (2021). Physical properties of porphyrin-based crystalline metal—organic frameworks. Commun. Chem..

[33] Figueira F., Paz F.A.A. (2021). Porphyrin MOF-Derived Porous Carbons: Preparation and Applications. C.

[34] Abdelhameed R.M., El-Shahat M., Abd El-Ghaffar M.A. (2022). Boosting the photocatalytic activity of Ti-MOF via emerging with metal phthalocyanine to degrade hazard textile pigments. J. Alloys Compd..

[35] Tsolekile N., Nelana S., Oluwafemi O.S. (2019). Porphyrin as Diagnostic and Therapeutic Agent. Molecules.

[36] Figueira F., Lourenço L.M.O., Neves M.G.P.M.S., Cavaleiro J.A.S., Tomé J.P.C. (2019). Synthesis and characterization of novel 5-monocarbohydrate-10,20-bis-aryl-porphyrins. J. Porphyrins Phthalocyanines.

[37] Castro K.A.D.F., Figueira F., Almeida Paz F.A., Tomé J.P.C., da Silva R.S., Nakagaki S., Neves M.G.P.M.S., Cavaleiro J.A.S., Simões M.M.Q. (2019). Copper-phthalocyanine coordination polymer as a reusable catechol oxidase biomimetic catalyst. Dalton Trans..

[38] Huang H., Song W., Rieffel J., Lovell J.F. (2015). Emerging applications of porphyrins in photomedicine. Front. Phys..

[39] Shi Y., Zhang F., Linhardt R.J. (2021). Porphyrin-based compounds and their applications in materials and medicine. Dyes Pigm..

[40] Figueira F., Cavaleiro J.A.S., Tomé J.P.C. (2011). Silica nanoparticles functionalized with porphyrins and analogs for biomedical studies. J. Porphyrins Phthalocyanines.

[41] Figueira F., Rodrigues J.M.M., Farinha A.A.S., Cavaleiro J.A.S., Tomé J.P.C. (2016). Synthesis and anion binding properties of porphyrins and related compounds. J. Porphyrins Phthalocyanines.

[42] Montaseri H., Kruger C.A., Abrahamse H. (2020). Recent Advances in Porphyrin-Based Inorganic Nanoparticles for Cancer Treatment. Int. J. Mol. Sci..

[43] Beg S., Rahman M., Jain A., Saini S., Midoux P., Pichon C., Ahmad F.J., Akhter S. (2017). Nanoporous metal organic frameworks as hybrid polymer–metal composites for drug delivery and biomedical applications. Drug Discov..

[44] Zhou Y., Liang X., Dai Z. (2016). Porphyrin-loaded nanoparticles for cancer theranostics. Nanoscale.

[45] Castro K.A.D.F., Figueira F., Mendes R.F., Almeida Paz F.A., Neves M.d.G.P.M.S., Cavaleiro J.A.S., Nakagaki S., Tomé J.P.C., Simões M.M.Q. (2019). Porphyrinic coordination polymer-type materials as heterogeneous catalysts in catechol oxidation. Polyhedron.

[46] Yang J., Yang Y.W. (2020). Metal-organic framework-based cancer theranostic nanoplatforms. View.

[47] Shao S., Rajendiran V., Lovell J.F. (2019). Metalloporphyrin nanoparticles: Coordinating diverse theranostic functions. Coord. Chem. Rev..

[48] Feng L., Wang K.-Y., Joseph E., Zhou H.-C. (2020). Catalytic porphyrin framework compounds. Trends in Chem..

[49] Ladomenou K., Natali M., Iengo E., Charalampidis G., Scandola F., Coutsolelos A.G. (2015). Photochemical hydrogen generation with porphyrin-based systems. Coord. Chem. Rev..

[50] Tian J., Huang B., Nawaz M.H., Zhang W. (2020). Recent advances of multi-dimensional porphyrin-based functional materials in photodynamic therapy. Coord. Chem. Rev..

[51] Pereira C.F., Simões M.M., Tomé J.P., Almeida Paz F.A. (2016). Porphyrin-based metal-organic frameworks as heterogeneous catalysts in oxidation reactions. Molecules.

[52] Qi Z.-L., Cheng Y.-H., Xu Z., Chen M.-L. (2020). Recent advances in porphyrin-based materials for metal ions detection. Int. J. Mol. Sci..

[53] Bavykina A., Kolobov N., Khan I.S., Bau J.A., Ramirez A., Gascon J. (2020). Metal–Organic Frameworks in Heterogeneous Catalysis: Recent Progress, New Trends, and Future Perspectives. Chem. Rev..

[54] Shan X., Zhang X., Wang C., Zhao Z., Zhang S., Wang Y., Sun B., Luo C., He Z. (2021). Molecularly engineered carrier-free co-delivery nanoassembly for self-sensitized photothermal cancer therapy. J. Nanobiotechnol..

[55] Algorri J.F., Ochoa M., Roldán-Varona P., Rodríguez-Cobo L., López-Higuera J.M. (2021). Photodynamic Therapy: A Compendium of Latest Reviews. Cancers.

[56] Pham T.C., Nguyen V.-N., Choi Y., Lee S., Yoon J. (2021). Recent strategies to develop innovative photosensitizers for enhanced photodynamic therapy. Chem. Rev..

[57] Algorri J.F., Ochoa M., Roldán-Varona P., Rodríguez-Cobo L., López-Higuera J.M. (2021). Light Technology for Efficient and Effective Photodynamic Therapy: A Critical Review. Cancers.

[58] Li X.-Y., Tan L.-C., Dong L.-W., Zhang W.-Q., Shen X.-X., Lu X., Zheng H., Lu Y.-G. (2020). Susceptibility and Resistance Mechanisms during Photodynamic Therapy of Melanoma. Front. Oncol..

[59] Kou J., Dou D., Yang L. (2017). Porphyrin photosensitizers in photodynamic therapy and its applications. Oncotarget.

[60] Yu W., Zhen W., Zhang Q., Li Y., Luo H., He J., Liu Y. (2020). Porphyrin-Based Metal-Organic Framework Compounds as Promising Nanomedicines in Photodynamic Therapy. ChemMedChem.

[61] Doughty A.C.V., Hoover A.R., Layton E., Murray C.K., Howard E.W., Chen W.R. (2019). Nanomaterial Applications in Photothermal Therapy for Cancer. Materials.

[62] Yu C., Xu L., Zhang Y., Timashev P.S., Huang Y., Liang X.-J. (2020). Polymer-Based Nanomaterials for Noninvasive Cancer Photothermal Therapy. ACS Appl. Polym. Mater..

[63] Hak A., Ravasaheb Shinde V., Rengan A.K. (2021). A review of advanced nanoformulations in phototherapy for cancer therapeutics. Photodiagn. Photodyn. Ther..

[64] Kim M., Lee J.H., Nam J.M. (2019). Plasmonic photothermal nanoparticles for biomedical applications. Adv. Sci..

[65] Wu M.X., Yang Y.W. (2017). Metal–organic framework (MOF)-based drug/cargo delivery and cancer therapy. Adv. Mater..

[66] Wang Z., Sun Q., Liu B., Kuang Y., Gulzar A., He F., Gai S., Yang P., Lin J. (2021). Recent advances in porphyrin-based MOFs for cancer therapy and diagnosis therapy. Coord. Chem. Rev..

[67] Chen J., Zhu Y., Kaskel S. (2021). Porphyrin-Based Metal–Organic Frameworks for Biomedical Applications. Angew. Chem. Int. Ed..

[68] Ni K., Lan G., Chan C., Quigley B., Lu K., Aung T., Guo N., La Riviere P., Weichselbaum R.R., Lin W. (2018). Nanoscale metal-organic frameworks enhance radiotherapy to potentiate checkpoint blockade immunotherapy. Nat. Commun..

[69] Lee G., Harnett N., Zychla L., Dinniwell R.E. (2012). Radiotherapy Treatment Review: A Prospective Evaluation of Concordance between Clinical Specialist Radiation Therapist and Radiation Oncologist in Patient Assessments. J. Med. Imaging Radiat. Sci..

[70] Rao J., Dragulescu-Andrasi A., Yao H. (2007). Fluorescence imaging in vivo: Recent advances. Curr. Opin. Biotechnol..

[71] Li C., Chen G., Zhang Y., Wu F., Wang Q. (2020). Advanced fluorescence imaging technology in the near-infrared-II window for biomedical applications. J. Am. Chem. Soc..

[72] Xue X., Lindstrom A., Li Y. (2019). Porphyrin-based nanomedicines for cancer treatment. Bioconjugate Chem..

[73] Liu M., Ren X., Meng X., Li H. (2021). Metal-Organic Frameworks-Based Fluorescent Nanocomposites for Bioimaging in Living Cells and in vivo. Chin. J. Chem..

[74] Grover V.P.B., Tognarelli J.M., Crossey M.M.E., Cox I.J., Taylor-Robinson S.D., McPhail M.J.W. (2015). Magnetic Resonance Imaging: Principles and Techniques: Lessons for Clinicians. J Clin Exp Hepatol.

[75] Vijayalaxmi, Fatahi M., Speck O. (2015). Magnetic resonance imaging (MRI): A review of genetic damage investigations. Mutat. Res. Rev. Mutat. Res..

[76] Geraldes C.F.G.C., Castro M.M.C.A., Peters J.A. (2021). Mn(III) porphyrins as potential MRI contrast agents for diagnosis and MRI-guided therapy. Coord. Chem. Rev..

[77] Imran M., Ramzan M., Qureshi A.K., Khan M.A., Tariq M. (2018). Emerging Applications of Porphyrins and Metalloporphyrins in Biomedicine and Diagnostic Magnetic Resonance Imaging. Biosensors.

[78] Calvete M.J.F., Pinto S.M.A., Pereira M.M., Geraldes C.F.G.C. (2017). Metal coordinated pyrrole-based macrocycles as contrast agents for magnetic resonance imaging technologies: Synthesis and applications. Coord. Chem. Rev..

[79] Seeram E. (2018). Computed Tomography: A Technical Review. Radiol. Technol..

[80] Lusic H., Grinstaff M.W. (2013). X-ray-Computed Tomography Contrast Agents. Chem. Rev..

[81] Fathi P., Pan D. (2020). Current trends in pyrrole and porphyrin-derived nanoscale materials for biomedical applications. Nanomedicine.

[82] Beard P. (2011). Biomedical photoacoustic imaging. Interface Focus.

[83] Prabhakar N., Rosenholm J.M. (2019). Nanodiamonds for advanced optical bioimaging and beyond. Curr. Opin. Colloid Interface Sci..

[84] Zhang Z., Zhang L., Wojtas L., Eddaoudi M., Zaworotko M.J. (2012). Template-Directed Synthesis of Nets Based upon Octahemioctahedral Cages That Encapsulate Catalytically Active Metalloporphyrins. J. Am. Chem. Soc..

[85] Zhang L., Lei J., Ma F., Ling P., Liu J., Ju H. (2015). A porphyrin photosensitized metal–organic framework for cancer cell apoptosis and caspase responsive theranostics. Chem. Commun..

[86] Zheng X., Wang L., Liu M., Lei P., Liu F., Xie Z. (2018). Nanoscale Mixed-Component Metal–Organic Frameworks with Photosensitizer Spatial-Arrangement-Dependent Photochemistry for Multimodal-Imaging-Guided Photothermal Therapy. Chem. Mater..

[87] Zheng X., Wang L., Guan Y., Pei Q., Jiang J., Xie Z. (2020). Integration of metal-organic framework with a photoactive porous-organic polymer for interface enhanced phototherapy. Biomaterials.

[88] Liu W., Wang Y.-M., Li Y.-H., Cai S.-J., Yin X.-B., He X.-W., Zhang Y.-K. (2017). Fluorescent Imaging-Guided Chemotherapy-and-Photodynamic Dual Therapy with Nanoscale Porphyrin Metal–Organic Framework. Small.

[89] Liu Y., Hou W., Xia L., Cui C., Wan S., Jiang Y., Yang Y., Wu Q., Qiu L., Tan W. (2018). ZrMOF nanoparticles as quenchers to conjugate DNA aptamers for target-induced bioimaging and photodynamic therapy. Chem. Sci..

[90] Chen Y., Liu W., Shang Y., Cao P., Cui J., Li Z., Yin X., Li Y. (2019). Folic acid-nanoscale gadolinium-porphyrin metal-organic frameworks: Fluorescence and magnetic resonance dual-modality imaging and photodynamic therapy in hepatocellular carcinoma. Int. J. Nanomed..

[91] He M., Chen Y., Tao C., Tian Q., An L., Lin J., Tian Q., Yang H., Yang S. (2019). Mn–Porphyrin-Based Metal–Organic Framework with High Longitudinal Relaxivity for Magnetic Resonance Imaging Guidance and Oxygen Self-Supplementing Photodynamic Therapy. ACS Appl. Mater. Interfaces.

[92] Zhang H., Tian X.-T., Shang Y., Li Y.-H., Yin X.-B. (2018). Theranostic Mn-Porphyrin Metal–Organic Frameworks for Magnetic Resonance Imaging-Guided Nitric Oxide and Photothermal Synergistic Therapy. ACS Appl. Mater. Interfaces.

[93] Alimoradi H., Greish K., Gamble A.B., Giles G.I. (2019). Controlled Delivery of Nitric Oxide for Cancer Therapy. Pharm Nanotechnol.

[94] Zhou G., Wang Y.S., Jin Z., Zhao P., Zhang H., Wen Y., He Q. (2019). Porphyrin–palladium hydride MOF nanoparticles for tumor-targeting photoacoustic imaging-guided hydrogenothermal cancer therapy. Nanoscale Horiz..

[95] Bao J., Zu X., Wang X., Li J., Fan D., Shi Y., Xia Q., Cheng J. (2020). Multifunctional Hf/Mn-TCPP metal-organic framework nanoparticles for triple-modality imaging-guided PTT/RT synergistic cancer therapy. Int. J. Nanomed..

[96] Wang C., Xiong C., Li Z., Hu L., Wei J., Tian J. (2021). Defect-engineered porphyrinic metal–organic framework nanoparticles for targeted multimodal cancer phototheranostics. Chem. Commun..

[97] Xie B.-R., Yu Y., Liu X.-H., Zeng J.-Y., Zou M.-Z., Li C.-X., Zeng X., Zhang X.-Z. (2021). A near infrared ratiometric platform based π-extended porphyrin metal-organic framework for O2 imaging and cancer therapy. Biomaterials.

[98] Yao S., Wang Z., Li L. (2022). Application of organic frame materials in cancer therapy through regulation of tumor microenvironment. Smart Mater. Med..

[99] Wan S.-S., Cheng Q., Zeng X., Zhang X.-Z. (2019). A Mn(III)-Sealed Metal–Organic Framework Nanosystem for Redox-Unlocked Tumor Theranostics. ACS Nano.

[100] Tian X.-T., Cao P.-P., Zhang H., Li Y.-H., Yin X.-B. (2019). GSH-activated MRI-guided enhanced photodynamic- and chemo-combination therapy with a MnO2-coated porphyrin metal organic framework. Chem. Commun..

[101] Zeng J.-Y., Zhang M.-K., Peng M.-Y., Gong D., Zhang X.-Z. (2018). Porphyrinic Metal–Organic Frameworks Coated Gold Nanorods as a Versatile Nanoplatform for Combined Photodynamic/Photothermal/Chemotherapy of Tumor. Adv. Funct. Mater..

[102] Zhang H., Li Y.-H., Chen Y., Wang M.-M., Wang X.-S., Yin X.-B. (2017). Fluorescence and Magnetic Resonance Dual-Modality Imaging-Guided Photothermal and Photodynamic Dual-Therapy with Magnetic Porphyrin-Metal Organic Framework Nanocomposites. Sci. Rep..

[103] Dhakshinamoorthy A., Navalón S., Asiri A.M., Garcia H. (2020). Gold-Nanoparticle-Decorated Metal-Organic Frameworks for Anticancer Therapy. ChemMedChem.

[104] Yin X., Ai F., Han L. (2022). Recent Development of MOF-Based Photothermal Agent for Tumor Ablation. Front. Chem..

[105] Zhu W., Liu Y., Yang Z., Zhang L., Xiao L., Liu P., Wang J., Yi C., Xu Z., Ren J. (2018). Albumin/sulfonamide stabilized iron porphyrin metal organic framework nanocomposites: Targeting tumor hypoxia by carbonic anhydrase IX inhibition and T1–T2 dual mode MRI guided photodynamic/photothermal therapy. J. Mater. Chem. B.

[106] Zhao M., Wang Y., Ma Q., Huang Y., Zhang X., Ping J., Zhang Z., Lu Q., Yu Y., Xu H. (2015). Ultrathin 2D metal–organic framework nanosheets. Adv. Mater..

[107] Zhao Y., Wang J., Pei R. (2020). Micron-Sized Ultrathin Metal–Organic Framework Sheet. J. Am. Chem. Soc..

[108] Schlachter A., Asselin P., Harvey P.D. (2021). Porphyrin-Containing MOFs and COFs as Heterogeneous Photosensitizers for Singlet Oxygen-Based Antimicrobial Nanodevices. ACS Appl. Mater. Interfaces.

[109] Sun Y., Jiang X., Liu Y., Liu D., Chen C., Lu C., Zhuang S., Kumar A., Liu J. (2021). Recent advances in Cu(II)/Cu(I)-MOFs based nano-platforms for developing new nano-medicines. J. Inorg. Biochem..

[110] Xu M., Yang S.S., Gu Z.Y. (2018). Two-dimensional metal-organic framework nanosheets: A rapidly growing class of versatile nanomaterials for gas separation, MALDI-TOF matrix and biomimetic applications. Chem. Eur. J..

[111] Zhu W., Yang Y., Jin Q., Chao Y., Tian L., Liu J., Dong Z., Liu Z. (2019). Two-dimensional metal-organic-framework as a unique theranostic nano-platform for nuclear imaging and chemo-photodynamic cancer therapy. Nano Res..

[112] Morais M., Ferreira V.F.C., Figueira F., Mendes F., Raposinho P., Santos I., Oliveira B.L., Correia J.D.G. (2017). Technetium-99m complexes of l-arginine derivatives for targeting amino acid transporters. Dalton Trans..

